# Effect of GaAlAs 940 nm Photobiomodulation on palatal wound healing after free gingival graft surgery: a split mouth randomized controlled clinical trial

**DOI:** 10.1186/s12903-022-02229-8

**Published:** 2022-05-24

**Authors:** Ghazal Morshedzadeh, Hoori Aslroosta, Mahsa Vafaei

**Affiliations:** 1 Department of Periodontics, School of Dentistry, Tehran University of Medical Sciences, Tehran, Iran; 2 Department of Restorative Dentistry, Babol University of Medical Sciences, Mazandaran, Iran

**Keywords:** Low level laser therapy, Free gingival graft, Wound healing, Biostimulation, Photobiomodulation

## Abstract

**Background:**

The aim of this study was to evaluate the effects of photobiomodulation (PBM) on wound healing, pain, and discomfort at free gingival graft (FGG) donor sites.

**Methods:**

Sixteen patients in need of bilateral FGG were selected for this randomized, controlled, triple-blinded, and split mouth clinical trial. The FGG donor sites in test group were treated with LLLT GaAlAs 940 nm, 5 J/cm^2^ immediately after surgery and every other day within the following ten days. The control group received sham irradiation. Remaining Wound Area (RWA), Epithelialization and color match were evaluated on the day of surgery and 7, 14, 21, 28, and 60 days after surgery. A questionnaire was administered to measure pain and bleeding in the first ten days after surgery.

**Results:**

RWA was significantly smaller in the test than control group on the days 7 (*p* < 0.001) and 14 (*p* = 0.048) after the surgery. Bleeding was higher in the test group than in the control group on the day of surgery (*p* = 0.046). Pain and discomfort at the palatal donor site, however, had no significant difference between laser and control group during 11 days after the surgery (*p* > 0.05), nor did the Color match scores on the 28th and 60th days after the surgery (*p* > 0.05).

**Conclusions:**

It can be concluded that PBM enhances FGG donor site wound healing one and two weeks after the surgery.

*Trial registration*

IRCT2017092036203N2, registered 01.11.2017.

## Introduction

Free gingival graft is among predominant augmentation treatment [1, 2] prescribed in order to increase the width of keratinized gingiva [3],increase the depth of vestibule [4], minimize the gingiva recession [5], and replace pigmented gingiva [6].

The long-term stability of the procedure has been well-proven for the above-mentioned areas of concern [7].

The graft involves the epithelium and a thin layer of the connective tissue and results in an open wound in the donor site. The wound heals by epithelialization in the form of a secondary healing within a period of two to four weeks [8], yet this triggers pain and discomfort both during and after the surgery; accordingly, some patients would rather demand a withdrawal from the process [9, 10].

What may also merit attention is that in case it is required to have a larger area of graft, the next surgery must be postponed until palate has completed its healing period.

Subsequently, should it be possible to devise a way so that the healing process could be accelerated and the pain could be mitigated, the surgery will be freed of a number of its major downsides.

A clot mostly consists of a fibrin network with inflammatory cells, red blood cells, and the debris left by the damaged cells making contributions to filling the gap between the soft tissue flap and the bone surface.

A myriad of both animal and clinical studies have been carried out to find ways/methods–low level laser therapy (referred to as LLLT here after on), local application of Ozonated oil [11], Platelet Rich Plasma [12], and Platelet Rich Fibrin [13] to mention but a few– for improving the healing of the palate wounds and reducing pain as well as surgical side effects.

Besides, how Anti-advanced Glycation End-product (AGE) factors [14] influence healing of the palate wounds and what side effects they are likely to cause have been demonstrated in rats [15].

LLLT using diode lasers of wavelengths 588–940 nm has several biostimulatory impacts on the target tissues; accordingly, the temperature at the target tissue is always well-calculated not to exceed the normal body temperature (98.6°F or 36.5 °C), so that the damage to the tissue could be as little as possible. [16, 17]

Both in medicine and dentistry, biostimulation enjoys a range of applications such as accelerating post-surgical wound healing period, recovery from dental diseases and reducing the inflammatory process. [17]

The main mechanism underlying biostimulation is based on the principles of photobiomodulation (PBM).

This is mainly associated with non-thermal impact of laser induced by its photochemical effect on cells [18–21].

Photobiomodulation (PBM) ameliorates the healing process through increasing the rate of movement in human keratinocytes, stimulating the primary epithelialization, rising the cell proliferation, improving the process of coagulation, developing matrices, and angiogenesis.

In addition, PBM can enhance the tensile strength and stability of soft tissue margins [22, 23]

There are, however, other studies not approving the impact of laser [24–27].

The contradictions in the results could be attributed to the discrepancies in treatment factors and limitations in the design of the studies such as comparing heterogeneous clinical wounds, absence of control groups, insufficient blinding of the subjects, and heterogeneity of the laser parameters [28, 29].

High concentrations of growth factors, cytokines, and hormones are responsible for regulating the process of wound healing.

When detecting a wound, the cells of innate immunity system excrete large amounts of ROS, acting as molecular messengers while they are transmitting cellular signals.

These free oxygen species have a dual function and can be beneficial or harmful depending on their concentration [30].

Recent studies have identified ROS as the main secondary messengers led by PBM.

Depending on the dosage, duration, and the level of radiation to wounds, LLLT regulates biochemical processes through uplifting the anti-oxidant system assigned to reduce tissue damages, boosting mitochondria breathing and producing ATP [31].

Most studies have failed to well demonstrate the effect of LLLT on improving the healing process as they selected improper parameters in laser radiation; as a result, there are controversies concerning the appropriate frequency and dosage of laser [17].

The biostimulatory effects of 940 nm GaAlAs laser on palatal donor site wound has been studied; [17, 32] The reduced number of inflammatory cells and the increased mitotic activity of fibroblasts were inferred after 4 sessions of LLLT at dose of 10 J/cm^2^ on full thickness palatal wounds in rats. Ustaoglu et al. in a controlled clinical study demonstrated the augmented healing of palatal wound after LLLT using 940 nm GaAlAs laser at dose of 8.6 J/cm^2^. Taking into account the subjective character of pain perception, it is recommended to be assessed in a split mouth study [32].

The aim of this split mouth randomized clinical study was to investigate the effect of PBM on accelerating post-surgical wound healing and reduction of pain and discomfort in palatal donor site, where 940 nm GaAlAs laser (0.21 w, 5 J/cm^2^) was used as a continuous wave.

## Methods and materials

### Participants

The study was a randomized, controlled, triple-blinded, and split mouth clinical trial, conducted on sixteen patients (16 test and 16 control) referred to the Periodontology Department of Tehran University of Medical Science, Tehran, Iran. The patients had a keratinized gingiva of less than 1 mm width on the required site and were nominated for gingival augmentation. Each participant provided written informed consent after receiving detailed verbal and written information about the study design.

The study was registered at the Ethics Committee of Tehran University of Medical Sciences and Iranian Registry of Clinical Trials (IRCT) web site (01/11/2017) holding the codes of IR.TUMS.DENTISTRY.REC.1396.3394 and IRCT2017092036203N2 respectively.

### Inclusion criteria

- Patients from both genders between the ages of 20 to 70, all systemically healthy
- Full Mouth Plaque Score (FMPS) of less than 20%
- Full Mouth Bleeding Score (FMBS) of less than 20%
- Keratinized gingiva of less than 1 mm width
- A need for gingival augmentation at two teeth located at two contralateral sides of the dental arch
- No pathologic or anatomic lesion at palate

### Exclusion criteria

The patients with the following issues were excluded from the study:

Active gag reflux, smoking habits, periodontal diseases, numbness, mobility and an occlusal trauma, extra forces such as mechanical forces generated by orthodontics and traumatic occlusion, systemic diseases considered either as the contra-indications of periodontal surgeries or in contradiction with wound healing process (such as diabetes), a history of treatment with high dose steroids, a history of taking anti-coagulation medications, radiotherapy or other types of therapies suppressing the immunity system, pregnancy, breast-feeding, digestive disorders, allergies to impression materials, a history of taking medications distorting the wound healing process or inducing gingival enlargement, and the patients missing the follow-up sessions after the surgery.

### Sample size

With regard to research published by Stephanie, Botti, Fernanandes, and Dias (2014) [33], the size of sample was 16 patients in each group; the proportion was one to one considering the power of 0.80734, alpha (0.05000) and beta (0.19266). The sample size was calculated using PASS software.

### Pre-surgical process

Prior to the surgical process, all patients received a professional periodontal prophylaxis phase. They also were given proper oral hygiene instructions.

The objectives of the instructions were to keep the soft tissues away from any trauma caused by improper brushing and flossing on one hand and to minimize the consequent biofilm and inflammation.

The patients were allowed to undertake the surgery only if their FMPS was below 20%. Meanwhile an impression of maxilla was prepared in order to make the acrylic stent required for protecting the palatal mucosa left by the surgical process.

### Surgical procedure

Based on the conventional procedure devised by Sullivan & Atkins, the surgical procedure of FGG was performed by a specialist unaware of participants’ groups.

In order to standardize the size and contour of the graft, a foil template made was prepared (14*9 mm).

Following a local anesthesia in both groups, the rectangular graft (1–1.5 mm thickness, with a surface area of 9*14 mm^2^) was harvested from the palate, within the area between the distal line angle of the canine and the mesial line angle of the maxillary first molar.

The FGG was sutured to the prepared recipient bed by using size 4–0 non-resorbable sutures.

To control bleeding and protect the palatal wound after the surgery, in the absence of any suture, the palatal acrylic resin stent was placed in each patient’s mouth and the patients were required to use it during the following week.

### Post-surgical instructions:

Patients received the essential instructions for not consuming hot and acidic meals and drinks.

Besides, for the pain and inflammation control, the patients were prescribed to take Gelofen (400 mg) every six hours for four days, Amoxicillin (500 mg) every 8 h and Chlorhexidine mouthwash (0.2%) twice a day for two weeks. The sutures of recipient site were removed after 2 weeks.

### Randomization and random allocation:

To comply with randomization, 16 sealed envelopes were designed, each containing one of the following phrases:

‘First-right-control’, ‘first-left-control’, ‘first-right-laser’, ‘first-left-laser’.

Following the bilateral surgery, an envelope was randomly selected so that the palatal laser radiation could be administered as instructed by the content of the envelope. Once the treatment chosen in the envelope was determined, the other side underwent the other type of treatment (split mouth). The patients were not informed of the content of envelops as well. The radiation was also carried out by a person rather than the one responsible for measuring the parameters.

### Radiation protocol

The wounds of donor site in the test group received the laser GaAlAs (epic X®) diode with the following parameters:

wavelength of 940 nm, power of 0.21 W, power density of 0.075 W/cm^2^, continuous mode, total dose of 6.3 J, wound area of 1.26cm^2^, fluence of 5 J/cm^2^, treatment sessions of 30 s, frequency of treatment 6 times (48 h intervals), probe spot size of 2.8 cm^2^. The power of equipment was calibrated by manufacturer, previously. In every session, the patient irradiated with a single application to the entire donor area.

Pain relief hand piece was used to radiate the laser beam 2–3 mm away from the wound surface. Table 1 shows the parameters of radiation on the test group. [17, 23, 34].

**Table 1:** Laser parameters

Manufacturer	Biolase
Model	epicX
Laser system	GaAlAs
Photobiomodulation probe	Whitening handpiece
Probe spot size	2.8 cm^2^
Wave length	940 nm
Power density	0.075 W/cm^2^
Power	0.21 W
Mode	Continuous wave
Application distance	2–3 mm (non-contact)
Fluence	5 J/cm^2^
Duration of each treatment session	30 Sec
Frequency of treatment	6 times (48 h interval)
Cumulative dose	6.3 J
Fiber optic	μ 400

### Blinding

In this study, the examiner, patients, clinical examiner, the person in charge of photograph analyses, and statistician were all blind (triple blinded), and merely was the laser operator aware of the radiation direction. For being blind, the examiners were required to make comments only about the final outcome, and their comments were all recorded. To keep the patients blind, the laser machine was in operation on both sides, yet the radiation was only applied to the test side, so that the patients could not detect the radiation side from the machine sound. On the control side, the machine was off free of any radiation.

### Variables and measurement method

#### Remaining wound area

Standard photographs of the surgical wound created on days 0, 7, 14, 28 and 60 using a CANON 70D. Wound area in terms of mm^2^ were measured using Image J—NIH software, Bethesda, USA and changes were recorded.

#### Epithelialization

Drippling hydrogen peroxide (3%) during the follow-up sessions (on the days 7th, 14th, 21st, 28th, and 60th after the surgery) made it possible to evaluate the bulbs forming on the surface of the wound. Occurrence of epithelialization was recorded in three groups namely: non-epithelialized, partially-epithelialized, and completely-epithelialized.

#### Pain and discomfort

After the surgery the patients were given a questionnaire to fill in (on the day of the surgery and the first 11 days after the surgery). and were required to express how much pain they felt on either side of the donor site; the questionnaire was based on the Visual Analogue Scale (VAS) in which ‘zero’ represents the absence of pain and ‘10’ corresponds to intolerable pain.

#### Bleeding

The same questionnaire also contained items to enquire the patients about the presence or absence of bleeding on either side of palate on the day of the surgery and during the first 11 days after the surgery.

#### Color match

The color match between the healed wound and healthy tissue around was measured by Adobe Photoshop CC2017 during the first and second months. It was analyzed based on the standard colorimetric parameters as well as the following formula: [35].

$$\Delta {\text{E}} = \, \left[ { \, \left( {{\text{L}}.{\text{wound }}{-}{\text{ L}}.{\text{adjacent}}} \right)^{2} + \, \left( {{\text{a}}.{\text{wound }}{-}{\text{ a}}.{\text{adjacent}}} \right)^{2} + \, \left( {{\text{b}}.{\text{wound }}{-}{\text{ b}}.{\text{adjacent}}} \right)^{2} } \right]^{1/2}$$

#### Statistical analysis

The quantitative variables in the test and control groups were analyzed by a Paired T-Test and the qualitative variables were measured by Wilcoxon Signed Ranks Test.

Significance level was *p* < 0.05.

## Results

**Fig. 1:**
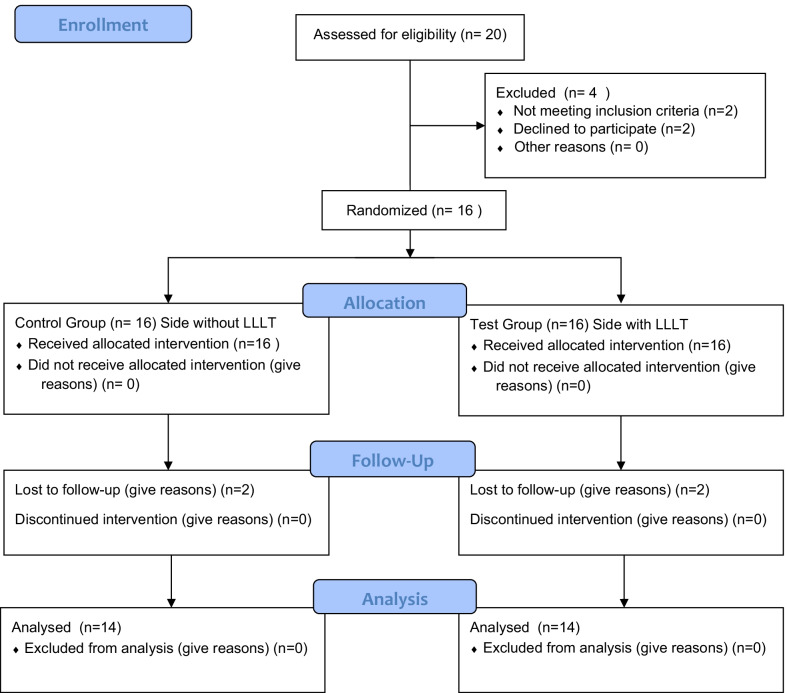
Flowchart of group divisions. LLLT, low-level laser therapy

In total 16 subjects received initial screening and baseline treatment. Two patients did not attend follow-up sessions and were excluded from the study (Fig. 1). 14 patients (10 females and 4 males) completed the study, aging between 29 to 65 years old (44 ± 10.3 in average).

### Remaining wound area

**Fig. 2:**
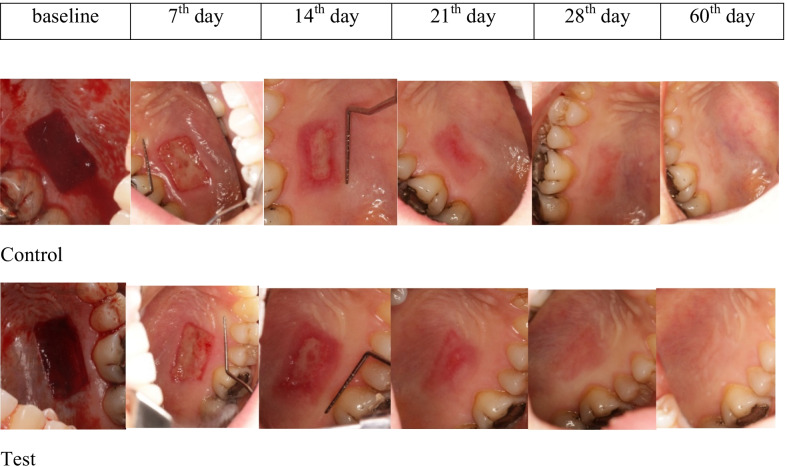
Clinical healing patterns of groups

On the 7th day, the initial area of the wound (134 mm^2^) experienced a reduction to 85.05 ± 27.88 mm^2^ on the laser side and 111.74 ± 27.31 mm^2^ on the control side (*p* < 0.001). On the day 14, the remaining wound saw another decline in size amounting to 20.34 ± 17.55 and 28.43 ± 20.85 on the laser and control sides respectively (*p* < 0.05). 28 days after the surgery, both groups enjoyed the total healing with no remaining wound. (Table 2 and Fig. 2).

**Table 2:** The mean remaining wound area in terms of mm^2^ on days 7 and 14 after surgery in each group

	Laser		control		*p*-value*
	Mean	SD	Mean	SD	
*Remaining wound area*					
Baseline	134.60	17.44	134.60	17.44	
Day 7	85.05	27.88	111.74	27.32	< 0.001*
Day 14	20.34	17.55	28.43	20.85	0.048*
Day 21	1.56	3.76	4.48	13.10	0.408
Day 28	0.00	0.00	0.09	0.33	0.336

*SD* standard deviation

^*^Paired T-test intergroup analysis

^*^p < 0.05, RWA was significantly smaller at laser group compared to control group at 7th and 14th days

### Epithelialization

On the 7th day, a larger number of patients on the control side were not epithelialized (10 patients in the laser group and 14 patients in the control group, *p* = 0.046). On the day 14, the figures had a rise on the laser side with a complete epithelialization (4 patients on the laser side and 1 patient on the control side, *p* > 0.05). The difference was not statistically significant though. Both groups had the complete epithelialization on the day 28. (Table 3 and Fig. 2).

**Table 3:** Number of donor sites with none, partial or complete epithelialization in laser and control groups at 7, 14, 21, and 30 days after surgery

Study Group	None		Partial		Complete		*p*-value*
	Laser	Control	Laser	Control	Laser	Control	
Day 7	10	14	4	0	0	0	0.046*
Day 14	2	1	8	12	4	1	0.414
Day 21	0	0	4	6	10	8	0.564
Day 28	0	0	0	0	14	14	0.317

^*^Wilcoxon signed ranks test: Test Versus Control groups

^*^p < 0.05, Significantly larger number of patients on the control side were not epithelialized on the 7th day

### Pain and discomfort

There was no statistically significant difference between the laser and control side in terms of pain (*p* > 0.05) (Table 4).

**Table 4:** Patient-reported pain on the test and control group in the first 11 days after surgery according to the VAS scale

	Laser		Control		*p*-value*
	Mean	SD	Mean	SD	
*Pain & Discomfort*					
Day 0	3.42	3.27	3.71	3.38	0.302
Day 1	2.42	1.74	2.78	2.08	0.136
Day 2	2.35	2.43	2.21	2.42	0.655
Day 3	2.50	1.87	2.57	2.50	0.888
Day 4	2.92	2.52	3.35	2.59	0.111
Day 5	2.64	3.10	3.14	3.39	0.151
Day 6	2.21	2.45	2.71	3.17	0.221
Day 7	2.71	2.33	2.5	3.03	0.150
Day 8	2.00	2.35	2.64	3.02	0.120
Day 9	1.57	1.91	2.21	2.66	0.179
Day 10	1.42	1.78	2.14	2.38	0.146
Day 11	0.85	1.29	1.42	2.17	0.205

*SD* standard deviation

^*^Paired T-test intergroup analysis without significant difference

### Bleeding

The secondary bleeding had no statistically significant difference between the laser and control groups, yet the level of bleeding was higher and statistically significant in the laser group on the day of the surgery (*p* = 0.046). on the second and third days after the surgery, bleeding was still more frequent with the laser side and six days after the surgery there was no bleeding in the patients (Table 5).

**Table 5:** Patient reported bleeding rate in the test and control groups in the first 11 days after surgery

		Laser	Control	P-value*
*Bleeding*				
Day 0	Bleeding	10	6	0.046*
	No bleeding	14	8	
Day 1	Bleeding	5	5	1.000
	No bleeding	9	9	
Day 2	Bleeding	4	2	0.157
	No bleeding	10	12	
Day 3	Bleeding	3	2	0.317
	No bleeding	11	12	
Day 4	Bleeding	2	2	1.000
	No bleeding	12	12	
Day 5	Bleeding	2	4	0.157
	No bleeding	12	10	
Day 6	Bleeding	0	0	1.000
	No bleeding	14	14	
Day 7	Bleeding	1	0	0.317
	No bleeding	13	14	
Day 8	Bleeding	1	1	1.000
	No bleeding	13	13	
Day 9	Bleeding	2	2	1.000
	No bleeding	12	12	
Day 10	Bleeding	1	1	1.000
	No bleeding	13	13	
Day 11	Bleeding	1	1	1.000
	No bleeding	13	13	

^*^Wilcoxon signed ranks test: Test Versus Control group

*p* < 0.05: The level of bleeding was higher and statistically significant in the laser group on the day of the surgery

### Color match

No statistically significant differences were observed between the groups (*p* > 0.05), yet the laser group enjoyed a better color match in both follow-up sessions than did the control group. (Table 6 and Fig. 2).

**Table 6:** Color matching on the test and control side during the first two months after surgery based on ΔE

	Laser		Control		*p*-value*
	mean	SD	Mean	SD	
Color match					
Day 28	15.85	9.91	17.48	8.53	0.659
Day 60	10.14	5.53	12.21	7.47	0.252

^*^Paired T-test intergroup analysis

## Discussion

Low-powered laser therapy may result in irreversible alterations in cells’ activities as well as tissues. The process, known as photobiomodulation (referred to as PBM here after on) may stimulate healing in periodontal wounds. [36].

The present study investigated the impact of PBM (as triggered by GaAlAs laser with the wave length of 940 nm, power of 0.21 W, and dosage of 5 J/cm^2^ radiated in a continues manner) on palatal wound healing, epithelization, post-surgical pain and discomfort, bleeding, and the wound tissue color match with that of the adjacent tissue.

The main objective of the study was to examine the effect of PBM on the wound healing of the palatal donor site once FGG harvesting had been removed accordingly.

Although many studies have been dedicated to measure the effect of PBM on acceleration of wound healing, there still remains a paucity of evidence in terms of how PBM may influence the healing of the palatal wounds and the optimal dose and treatment protocol required to do so [33].

Nevertheless, it has been shown that there exist direct relationships between the optimal dose and the treatment target (mitigating the pain, accelerating the healing process, and regenerating the tissues) the depth of the target tissue, absorption characteristics of target tissue, the type of laser, the wave length, the density of power (irradiance), the density of energy (fluence), the duration and frequency of radiations, and the difference between the laser spot size and the surface area of the target tissue [37].

In order for PBM to occur efficiently, irradiance must be at a certain level, with the density not exceeding the thresholds at which the energy of photons is likely to create excessive heat within the target tissue, while a low-powered density may reduce the absorption of photons, falling below the required level.

In other words, Arndt–Schultz law can account for the biphasic response to the laser in PBM. Although there has been no consensus on the optimal area of fluence and irradiance to achieve the window effect, a myriad of studies has concluded that the fluence of 3–10 J/cm^2^ is able to boost the metabolic activity at the cellular level [38].

Further, the depth of the wound can be also influential on the value of the optimal density. By the way of illustration, some studies have suggested that the optimal doses for the shallow and deep tissues are 1–10 and 10–50 J/cm^2^ respectively [37]. Accordingly, as the palatal wound in the present study was of a shallow nature, developed by FGG harvesting, the authors decided to opt for the dose of 5 J/cm^2^ in agreement with the above mentioned studies. The tissue penetration is affected by the laser wavelength. While shorter wavelengths (600–700 nm) are preferred to treat superficial tissues, longer wavelengths (780 − 950 nm) are mostly approved for deeper tissues [39–41]. This could be related to the Mie scattering within tissue which is more intense for shorter wavelengths [42]. Although, lasers in the range of red light or near-infrared (600–1100 nm), are the mostly used for PBM, the optimum wavelength is considered to be 810 nm. However, wavelengths ranged to 950 nm are required to target the cutaneous and deeper tissues. Near-infrared (NIR) wavelength and red wavelength penetrate 2 mm and 0.5 − 1 mm in order before losing 37% of intensity [43]. In addition to the wavelength, the photobiomodulatory effects of diode lasers are related to their absorption in melanin (in basal cell layer and upper layers of palatal mucosa) and hemoglobin, resulting in improved outcome in depths of 5–10 mm. Studies on tissues with lower number of mitochondria (like skin) have found positive outcome are mostly attained with higher fluence values [42] while, negative studies mostly used lower fluences.

The palatal wound subsequent to FGG harvesting is an acute surgical wound healing by secondary intention. Normally acute wounds heal thorough four overlapping stages (hemostasis, inflammation, proliferation and remodeling) within 3 weeks [44]. After palatal graft harvesting, epithelial cells start to migrate within 24–48 h from the wound edges. Wound epithelialization complete after the third to the fourth week if left unaided [45, 46]. The main goal of lasers used on the wound management is to improve the microenvironment to reinforce the cellular activity at the wound area. In this study, faster epithelialization in laser group was seen after 7 and 14 days. In the context of the laser biostimulatory effect on the proliferative and secretory functions of cells, Gabriel found that PBM therapy on surgical oral ulcer in the dorsum of rat tongue, using 660 nm continuous wave diode laser with energy density of 4 J/cm^2^ applied for 10 consecutive day upregulated expression of NF-κB in keratinocytes on day 3 and downregulated it on day 10 which resulted in increased proliferation of keratinocytes in the early stage and differentiation in the last stages of wound repair [47].

### Remaining wound area (RWA)

The results of the study showed that RWA on the laser site was significantly lower than that of the control side after 7 and 14 days. Prior to our study, two other projects applied a diode laser (the wave length of 940 nm) to examine PBM of the palatal wound. Ustaoglu et al. conducted a randomized controlled clinical experiment and arrived at the conclusion that LLLT could enhance wound epithelization and secondary wound healing in the palatal donor site. This was in total agreement with the results of the present study [32].

Firat et al. investigated the impact of low-powered laser therapy (GaAlAs 940 nm) on palatal wound healing in diabetic rats. Histopathologic findings of the study indicated a reduction in the number of inflammatory cells in 7 days, a rise in mitotic activities among fibroblasts, generation of collagen on the days 14 and 21. After two weeks, the laser group enjoyed a corneum layer formed with a mature structure. Thus, it was concluded that LLLT had a positive effect on both the inflammatory response and the growth in proliferation of fibroblasts, being able to heal the palatal mucosal wound through stimulation and vascularization as well as generation of collagen [17].

Almeida et al. examined how low-powered laser might affect the donor site in FGG, reporting no statistically significant difference between the test and the control groups in terms of clinical wound healing [48]. Marcel et al. also focused on beagle dogs to see the effects of laser therapy on wound healing. From the microscopic view, they did not observe any statistically significant difference between the laser and control group in their wound healing levels [49]. These two differed with what we did in their radiation parameters. Table 7 shows the radiation parameters in different studies. While we relied on the density of 5 J/cm^2^, Almeida et al. and Marcel et al. used 10 J/cm^2^ and 1 J/cm^2^ respectively. The other dissimilarity was due to the way the wound healing was examined. Almeida et al. relied on skilled periodontists to examine the photographs, whereas Marcel et al. performed the clinical examination by measuring two spots tattooed (after the surgery) at a one-millimeter distance from the wound margins on the palatal mucoperiosteum. However, we measured RWA by Image-J software, run to examine the photographs captured in a standard way with higher accuracy than visual observations [48, 49].

**Table 7:** Characteristics of included studies

Study	Model	System	Probe spot size	Wave length	Power	Power density	Mode	Fluence	Frequency of treatment	Duration of each emission
Present study	epicX	GaAlAs	2.8 cm^2^	940 nm	0.075 W/cm^2^	0.21 W	Continuous mode	5 J/cm^2^	6 times (48 h interval)	30 Sec
Ustaoglu et al. [32]	Ezlase	GaAlAs	2.8 cm^2^	940 nm	1.07 W/cm^2^	3 W	Continuous mode	8.6 J/cm^2^	4 times (48 h interval)	8 Sec
Heidari et al. [46]	N/A	Diode laser	0.5 cm^2^	660 nm	N/A	0.2 W	Continuous mode	32 J/cm2 (4 J/cm^2^ per point and an application time of 4 s per point)	5 times (immediately after surgery and 1, 2, 4 and 7 days later)	32 Sec
Dias et al. [33] (CTG)	N/A	GaAlAs	N/A	660 nm	N/A	0.03 W	Continuous mode	15 J/cm^2^ (3 J/cm^2^ per point and an application time of 4 s per point)	8 times (48 h interval)	20 Sec
Ozcelic et al. [50] Gingivectomy)				588 nm		0.12 W	Continuous mode	4 J/cm^2^	8 times (immediately after surgery and daily for 7 days)	5 min
Almieda et al [48] (recipient site)	Twin Laser, MM Optics Ltda	GaAlAs		780 nm (an infrared wavelength to achieve analgesia) and also at 660 nm (a red wavelength to accelerate the healing)		0.04 W	Continuous mode	10 J/cm^2^	2 times (during the immediate postoperative period and 48 h later)	20 Sec/site
Marcel et al. [49] (animal study)		GaAlAs		830 nm		0.03 W			10 times (immediately after surgery and further three times a week)	33 Sec
Koo et al. [51] (animal study)		Diode laser		660 nm		0.06 W		1–4 J/cm^2^	T1(received laser treatment for 1 day) T3(received laser treatment for 3 days) T5(received laser treatment for 5 days)	20 min
Firat et al. [17] (animal study)	Ezlase	GaAlAs	0.09 cm^2^	940 nm	1.11 W/cm^2^	0.1 W	Continuous mode	10 J/cm^2^	4 times (first dose of irradiation at 2 h after the wounding procedure and were subsequently irradiated at 2-day intervals following the surgery for a total of four sessions)	9 Sec

### Epithelialization

In wound healing by the secondary intention type, epithelialization is of a pivotal importance; healed wound calls for the surface to be thoroughly covered by epithelium [46]. The authors, therefore, decided to employ hydrogen peroxide (3%) to examine the levels of epithelialization in the laser group, revealing a significantly higher level on the day 7 (*p* = 0.046). Evaluating the impact of diode laser on the levels of post-surgical epithelialization (after FGG harvesting) in palatal wounds, Heidari et al. found that the levels were significantly higher in the laser groups on the days 14 and 21, with no statistically significant differences on the day 7 though [46]. The discrepancy between the results might be explained by the differences in the radiation parameters and the examiners’ points of view.

The results we achieved were also different with that of Ustaoglu et al. as they reported the total epithelialization of the laser group on the 14, whereas we did not record any statistically significant difference on the days 14 and 21, while as indicated by the table of frequency the healing process was clinically in favor of the laser group; this might be in the shade of the small sample size in our study [32].

### Pain and discomfort

Ozcelic et al. concluded that LLLT could ease the post-surgical pain and discomfort in FGG harvesting [50]. In our study, both groups complained of gradually declining levels of pain not significantly different. Even though, the laser group had more pain on the days 2 and 7, they experienced less pain on other days. Koo carried out a study to examine the effect of the low-powered laser on reducing the pain of skin wounds in the rats [51]. The results showed that the laser radiation with the afore-mentioned parameters decreased the c-ƒos marker (that is the stimulator of nociceptive neurons in dorsal root of spinal cord and resulted in a significant decline of pain. The radiation parameters Koo applied differed from what we relied on; besides, in contrast with Koo’s biochemical and western blot methods, we measured the variable of pain by both a rather subjective way of asking for the patients’ comment and VAS scale, increasing the risk of error and bias.

### Bleeding

PBM results in vasodilation, a reduction in the contraction of smooth muscle cells in the walls of blood vessels, and a subsequent localized blood flow; this increases the cellular supplying oxygen and migration of immune cells to tissues [52–54]. As a result, it is expected to see a rise in the rate of wound bleeding, triggered by the laser radiation right before the process of coagulation. The same was true about the present study where on the day of surgery the laser group underwent a significantly higher bleeding rate than that of the control group. Heidari did not observe any significant differences in terms of both immediate and delayed bleeding, while there was an immediate bleeding right after the radiation of low-powered laser in two cases [46]. Thermographic studies have signified that laser treatment indirectly increases the tissue temperature and blood flow, ranging from 0.9 to 4 degrees. Ustaoglu et al. patients were reported with a significant lower rate of bleeding on the surgical site during the first two days after the surgery [32]. Since, except for one study reporting the improving effect of laser on the pre-coagulation activity of plackets [55], no studies have reported the anti-coagulation effect of laser therapy, this might have justified the results attained by Ustaoglu et al. Therefore, regarding the discrepancies in the results and the limitations surrounding the studies on the effect of laser therapy on the bleeding in the donor site, further studies need to be conducted to well address the area.

### Color match

There was no statistically significant difference between the groups with respect to their color match (*p* > 0.05), the laser group experienced a better color match in both follow-ups though. Both groups clinically enjoyed a higher level of color match in the second month.

In da silva’s study, the color match between the healing tissue and the surrounding one was considered as a criterion to examine inflammatory process [56]. Accordingly, both laser groups had a considerably better color match than control group on the day 14. In the present study the groups did not have any statistically significant differences in their color match during the first two months; this could be explained by the small sample size as the laser group had a clinically better color match. Another factor at play might be the difference in measurement timing (7th, 14th, 45th, 60th, 90th day) and the type of surgical procedure (sub-epithelial connective tissue graft harvested for root coverage and 4–0 silk sutures on donor sites) in two studies.

Ustaoglu et al. showed that the laser side had significantly better color match levels than what observed on the control side on the days 3, 7, and 14(32). Beside the different radiation parameters, Ustaoglu et al. worked on a larger sample size, investigating the color match by a periodontist’s direct observation as well as VAS scale, while we applied the light/optical parameters in Photoshop with higher levels of accuracy in comparison with what they did.

### Limitations

Among the limitations of the present study was the way the variable of pain and discomfort was measured: first, the study was conducted in a split mouth manner with four wounds in the patient’s mouth (two in the palatal donor site and two in the recipient region in mandible). The large number of the wounds increases the risk of referral pain and not recognizing the accurate levels of pain. Second, the restoration of FGG in the donor site was of a secondary intention in nature; consequently, the wound and neural terminals being exposed made it more difficult to differ the groups [56]. Next, our study was subject to bias and errors as we sufficed to measure the variable of pain in a rather subjective way through a questionnaire along with VAS scale. Further, in accordance with the post-operative protocol, once operated, all patients were prescribed with NSAID, influencing their sense of pain and discomfort in return. In the present study, the primary goal and outcome were acceleration of wound healing and reducing RWA; the authors were forced to choose the radiation protocol and the sample size accordingly. Nevertheless, larger sample size probably better clarifies the statistical difference between treatment modalities.

The patients in the study received the low-powered (GaAlAs 940 nm) laser radiations (six times) during a ten-day period on an every-other-day basis. That frequent patients’ referrals (to receive the laser radiations) made them available to be constantly under supervision. The moot question, however, is as to whether the same number of patients would carry the burdens of costs and commute to receive the treatment out of a research ambiance, and how far the protocol would be clinically efficient and practical. Thus, it is desirable to find a laser radiation protocol in need of less time, lower costs and fewer times of clinical referrals, bringing about patients’ cooperation so that laser therapy can turn to a more popular method of treatment.

As dictated by the limitations, it is recommended to perform further random controlled experimental clinical studies and objectively address a larger sample size, with histologic assessments and evaluations of pain-related factors. This may help to find the best radiation parameters, accelerating the healing process and simultaneously mitigating the patients’ post-operative pain and discomfort beside other secondary outcomes as observed in the present study.

## Conclusion

The limitations of the present study made us conclude that applying low-powered laser (GaAlAs 940 nm) with the selected parameters contributes to acceleration of donor site wound healing and reduction of RWA during the first and second weeks after the FGG surgery. It also increases the wound epithelialization in the palatal donor site a week after the surgery. The dosage results in further bleeding on the day of surgery though. No improvement in pain relief was obtained using this laser setting.

## Data Availability

The datasets generated and/or analysed during the current study are not publicly available due [Potential proprietary] but are available from the corresponding author on reasonable request.
